# Flipping the switches: CD40 and CD45 modulation of microglial activation states in HIV associated dementia (HAD)

**DOI:** 10.1186/1750-1326-6-3

**Published:** 2011-01-11

**Authors:** Jon Salemi, Demian F Obregon, Anthony Cobb, Spenser Reed, Edin Sadic, Jingji Jin, Francisco Fernandez, Jun Tan, Brian Giunta

**Affiliations:** 1 Department of Psychiatry and Neurosciences, Neuroimmunology Laboratory, University of South Florida, College of Medicine, Tampa, FL 33613, USA; 2 Department of Psychiatry and Neurosciences, Rashid Developmental Neurobiology Laboratory, Silver Child Development Center, University of South Florida, Tampa, FL 33613, USA; 3 Department of Molecular Medicine, University of South, College of Medicine, Tampa, FL 33613, USA

## Abstract

Microglial dysfunction is associated with the pathogenesis and progression of a number of neurodegenerative disorders including HIV associated dementia (HAD). HIV promotion of an *M1 *antigen presenting cell (APC) - like microglial phenotype, through the promotion of CD40 activity, may impair endogenous mechanisms important for amyloid- beta (Aβ) protein clearance. Further, a chronic pro-inflammatory cycle is established in this manner. CD45 is a protein tyrosine phosphatase receptor which negatively regulates CD40L-CD40-induced microglial *M1 *activation; an effect leading to the promotion of an *M2 *phenotype better suited to phagocytose and clear Aβ. Moreover, this CD45 mediated activation state appears to dampen harmful cytokine production. As such, this property of microglial CD45 as a regulatory "off switch" for a CD40-promoted *M1*, APC-type microglia activation phenotype may represent a critical therapeutic target for the prevention and treatment of neurodegeneration, as well as microglial dysfunction, found in patients with HAD.

## The Role of Microglia in HIV Associated Dementia (HAD)

**Figure 1:**
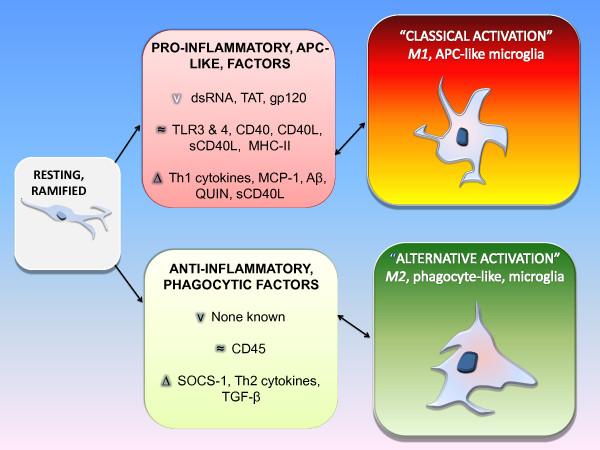
**Modulation of Microglia Phenotypes in HIV associated dementia (HAD)**. The roles of microglia include phagocytosis, antigen presentation, as well as generation and excretion of cytokines, eicosanoids, complement components, and excitatory amino acids (EAA) including, glutamate, quinolinic acid (QUIN), oxidative radicals, and nitric oxide (NO) [2]. At least three phenotypic states of microglia exist based on developmental and pathophysiologic studies: (*i*) resting, ramified; (*ii*) activated non-phagocytic (or APC like) found in areas involved in central nervous system (CNS) inflammation; and (*iii*) reactive, phagocytic micorglia observed in areas of trauma or infection [3-7]. In regard to activation, these cells are able to polarize into two major subtypes: *M1 *or *M2 *[8,9]. *M1 *subtype over-produces pro-inflammatory cytokines. It is marked by production of high levels of interferon -gamma (IFN-γ) tumor necrosis factor (TNF)-α, interleukin (IL)-1, IL-12, and low levels of IL-10 [8,9]. The *M1 *phenotype may be activated when microglia contact HIV proteins (such as transactivator of transcription [Tat]) [10] or bind toll-like receptors 3 or 4 as well [11]. *M2 *microglia dampen inflammation, become phagocytic, and produce very low levels of TNF-α, IL-1, IL-12 and high amounts of anti-inflammatory IL- 10 and transforming growth factor (TGF)-β, and SOCS (suppressor of cytokine signaling) [12,13]. These two phenotypes, respectively, correspond to the type *ii *or *iii *microglial states described in the preceding paragraph. Further, the factors which cause polarization to *M1 *or *M2*, reinforce the maintenance of that phenotype in a cycle-like manner [8,9] (Figure 1). Increased *M1 *polarization is consistent with increased TNF-α observed in plasma and brain specimens in HAD and AD, and may play a role in the pathophysiology of both diseases [14]. Stimulation of Th1 and Th2 immune response by microglia is dependent upon the expression of specific molecules including major histocompatibility complex (MHC) II and CD40 [15]. v = viral factors ~ = soluble or cell surface receptor ligation Δ = cytokines and soluble factors

Macrophages and microglia compose some 12% of the cells in the central nervous system (CNS) [1]. Their roles include phagocytosis, antigen presentation, as well as generation and excretion of cytokines, eicosanoids, complement components, and excitatory amino acids (EAA) including, glutamate, oxidative radicals, and nitric oxide (NO) [2]. At least three phenotypic states of microglia exist based on developmental and pathophysiologic studies: (*i*) resting, ramified; (*ii*) activated non-phagocytic (or APC like) found in areas involved in central nervous system (CNS) inflammation; and (*iii*) reactive, phagocytic microglia observed in areas of trauma or infection [3-7] (Figure 1).

In regard to activation, macrophages and microglia are able to polarize into two major subtypes, categorized as *M1 *or *M2 *[8,9]. The "classical" or *M1 *subtype over-produces pro-inflammatory cytokines and promotes cell-mediated immunity [8,9]. It is marked by production of high levels of interferon -gamma (IFN-γ), tumor necrosis factor (TNF)-α, interleukin (IL)-1, IL-12, and low levels of IL-10. The *M1 *phenotype may be activated when microglia contact HIV proteins (such as transactivator of transcription [Tat]) [10] bind toll-like receptors 3 or 4 as well [11]. "Alternatively activated" or *M2 *microglia tend to dampen inflammation, clear cellular debris (including amyloid plaques), and produce very low levels of TNF-α, IL-1, IL-12 and high amounts of anti-inflammatory IL- 10 and transforming growth factor (TGF)-β, and SOCS (suppressor of cytokine signaling) [8,9,12,13]. These two phenotypes, respectively, correspond to the type *ii *or *iii *microglial states described in the preceding paragraph. Further, the factors which cause polarization to *M1 *or *M2 *reinforce the maintenance of that phenotype in a cycle-like manner (Figure 1). Increased *M1 *polarization is consistent with increased TNF-α observed in plasma and brain specimens in HAD and AD, and may play a role in the pathophysiology of both diseases [14].

Stimulation of Th1 and Th2 immune response by microglia is dependent upon the expression of specific molecules including major histocompatibility complex (MHC) II and CD40 [15]. Microglia expressing MHC II induce CD4+ T cells to generate IFN-γ and TNF-α [16]. In the case of both HAD and AD, this response is considered harmful to the brain and in both diseases TNF-α is elevated to neurotoxic levels while only in HAD is IFN-γ is prominently elevated [14].

In HIV associated dementia (HAD; also known as NeuroAIDS, or HIV encephalitis [HIVE]), microglia and macrophages are productively infected by HIV-1 and show diffuse inflammatory activation, which ultimately leads to neuronal damage, death, CNS dysfunction [17,18]. A clinical trial using a small number of *post-mortem *HIV-infected individuals showed a direct correlation between microglial activation/infection and cognitive decline [19]. Studies have found microglial HIV infection as central in exacerbating HIV dementia [20,21]. Importantly, neuronal dysfunction and death in HIV infection results from cytokine stimulation, but especially several cytokine-mediated apoptotic mechanisms emanating from microglia. Thus microglial cytokine production is central to the pathogenesis of HAD [22,23].

Indeed, viral infection and/or immune activation of microglia fuels HAD pathogenesis ending in neuronal-injury and death [24,25]. Microglia are the main target for the HIV-1 infection in the brain. The virus infiltrates the CNS *via *infected monocytes [26,27]. Once infected or activated by HIV- proteins such as gp120 or Tat, microglia begin to excrete endogenous pro-inflammatory cytokines of the *M1 *subtype [28].

Histopathologically, activated microglia represent a highly accurate correlate to neuronal death and damage in CNS [29]. Severity of dementia in persons with HAD is strongly correlated with the number of activated macrophages and microglia within the basal ganglia and frontal lobes [30,31]. Moreover, activation of microglial cells by HIV is associated with astrogliosis, myelin pallor, and severe neuronal loss [24,30]

Recently, with the advent of highly active antiretroviral therapy (HAART) patients with HIV have been living significantly longer lives. While HAART has been increasing the lifespan of those infected with HIV, it has also led to an increased prevalence of HAD [32-38]. As the pathology of HAD, like Alzheimer's Disease (AD), is commonly characterized by an increase in the amount of amyloid-beta (Aβ) peptide in the brain [39], evidence suggesting microglia modulate the clearance of potentially neurotoxic Aβ species from the brain is of special importance [40,41].

Indeed, microglia play a major role in the neuropathogenesis of HAD and AD in quite similar ways, although the etiology of these diseases differ greatly [14]. Neuropathological similarities between HAD and AD include cortical neuronal loss and amyloid plaque deposition [39,42-44]. Indeed, most forms of dementia are accompanied by a widespread degeneration in the cerebral cortex - such as the plaques in AD brain. AD is thus considered a "cortical dementia." HAD is also considered to be a cortical dementia however there is also targeted damage to regions lying under the cortex. Some authors consider HAD to be a subcortical dementia however this terminology is somewhat inaccurate. HAD can cause damage to both cortical and subcortical areas. The resulting brain damage is often visualized on MRI as generalized brain atrophy and also visibly damaged subcortical areas [45,46].

Amyloid plaques in AD result from the deposition of amyloid beta (Aβ) which is a putative pathogenic molecule in AD. Aβ is the cleavage product of the amyloid precursor protein (APP) and APP mutations are associated with inherited forms of AD. The clinical implication or pathogenic consequences of brain amyloid deposition are still controversial in the AD field; although, the finding of Aβ deposition in both AD and HAD strongly suggests parallel pathways of chronic inflammation-mediated change that eventually yields cortical dysfunction characterized by identical "biomarkers". For example, decreased cerebrospinal fluid (CSF) Aβ and increased tau (a component of the neurofibrillary tangle, a second AD neuropathological hallmark) have been proposed as sensitive and specific markers of AD in several studies [47,48]. It has also been found that changes in CSF Aβ and tau are comparable to those observed in AD and HAD patients [49]. The pathogenic significance of these biomarkers is not well established but it has been hypothesized that decreased CSF Aβ indicates increased aggregation of insoluble Aβ and sequestration into amyloid plaques [50].

The mechanisms of neurodegeneration, which are highly microglia-dependent, in AD and HAD are similar in many ways as well [14]. Cascades of inflammatory processes lead to neurodegeneration in both dementias. The initial step in each disease differs. HAD is secondary to infection with HIV-1, while the exact cause of AD remains to be established. A common feature among both diseases is the interactions of microglia which promote a neurotoxic inflammatory environment. These interactions play significant roles in the initiation and continuation of the neurodegenerative process in each disease [14].

In both diseases, whether activation is by HIV itself, its proteins, or Aβ peptides, microglia release cytokines, reactive oxygen species (ROS), and several neurotoxins that impair cellular function, neurotransmitter action, and induce neuronal loss [51,52][14,53]. Some of these neurotoxins in both forms of dementia include TNF-α, arachidonic acid, platelet activating factors (PAF), nitric oxide (NO), and quinolinic acid (QUIN) [17,53-59]. Nitric oxide is synthesized by endothelial cells, neurons, and macrophages and is thought to be associated with NMDA-type glutamate-initiated neurotoxicity [54].

TNF-α is released by HIV-1-infected microglia, and oligodendrocytes are particularly sensitive to its effects [60]. Steady-state levels of TNF-α mRNA are higher in the subcortical regions of the CNS of patients with HAD than in HIV-1-infected patients without CNS involvement [61]. QUIN is a highly excitotoxic marker most well known in HIV neurological disease which may reflect the extent of immune activation in both blood and the brain and correlates with systemic and neurological disease status [17,53,55-59].

During immune activation, particularly while levels of IFN-γ are increased, induction of the enzyme indoleamine 2,3-dioxygenase occurs, increasing the synthesis of QUIN [53,62-64]. HIV-infected microglia also release chemokines [65], which may enhance infiltration and recruitment of both infected and uninfected microglia [53].

HIV encephalitis is typically marked by the presence of multinucleated giant cells and microglial nodules by immunohistochemistry or *in situ *hybridization. The presence of microglia in the CNS is strongly associated with severe neurobehavioral complications [66-69]. Microglia, as a major target of HIV-1 infection in the CNS, are typically a viral reservoir [70-72] and are also key in HIV-1 neuroinvasiveness-penetration into the CNS by the virus [72,73]. Most importantly, a discrepancy between the localization of HIV-infected cells and the severity of neurocognitive symptoms has been described [74-76]. Thus, other mechanisms secondary to virus infection, such as passage of monocytes and lymphocytes into the brain, activation of astrocytes/microglia, and production and release of inflammatory cytokines, all participate in the pathogenesis of HAD. This is a key concept which makes the neuropathogenesis of HAD, in many ways, similar to that of AD.

β-amyloid is a potent and direct neurotoxic agent [77-79], much like the HIV-1 proteins gp120 and Tat, and it induces a cascade of cellular mechanisms including activation of microglia [80], which leads to neuronal damage [81]. Indeed, reactive microglia are closely associated with neuritic and β-amyloid plaques, just as they are with HIV-1 Tat protein [82-89]. Using electron microscopic techniques, interactions between microglia and astrocytes have been observed [90], which may be associated with the production of cytokines that are also over-produced in the HAD brain such as IL-1β, tumor TNF-α, complement proteins, and ROS [81,91-94]. Research by our group and others of the microglia signal transduction pathways mediating the neurotoxic response of Aβ demonstrated that mitogen-activated protein-kinase (MAPK) superfamily members ERK1/2 and p38 MAPK act as mediators [95-97]. Furthermore, several lines of evidence indicate the NF-κB in microglia is stimulated by β-amyloid [98,99]. Activation of NF-κB can stimulate transcription of genes expressing TNF-α, IL-1, IL-6, monocytes chemo-attractant protein-1(MCP-1), and nitric oxide synthase (NOS). This too is re-capitulated in HAD as several lines of evidence indicate HIV gp120 and Tat activate the same pathway, leading to the production of the same neurotoxins [88,89,100-103].

Adding biological "insult to injury," in the HIV-1 infected brain, microglial phagocytosis of Aβ_1-42 _peptide appears inhibited [35]. The deposition of Aβ plaques in the HIV-1 infected brain is likely caused by several factors including the effects of cytokines and HIV-1 proteins on microglial phenotype, activation and activity. IFN-γ is hypothesized to enhance the effects of HIV-1 Tat by promoting the switch from a microglial phagocytic phenotype to one that is an antigen presenting cell (APC) phenotype [37].

## Modulation of Microglial activation in HAD: CD40, CD40L, sCD40L and CD45

CD40L is a 33-kDa type II membrane glycoprotein that is predominantly expressed by activated T cells, B cells, myeloid cells, and platelets. It has been well established that CD40L upregulates the immune response by leading to increased CD4+ T cell activation; an effect which promotes the replication of HIV in infected lymphocytes and immune cells [104] and also that robust CD40 ligation promotes an inflammatory and neurotoxic environment in the brain [105,106].

Elevated levels of sCD40L are found in an array of neurodegenerative diseases including HAD, AD, and multiple sclerosis (MS) [106]_. _This soluble protein is thought to initiate or potentiate an inflammatory cycle [106-109] in these conditions. Indeed, inflammation upregulates expression of CD40 receptor on the surface of endothelial cells and the shedding of the ligand [110]. Inhibition of CD40-CD40L interactions was shown to retard the development of experimental autoimmune encephalomyelitis (EAE), in an animal model of MS [111]. *In vitro *studies demonstrated IFN-γ, which is overexpressed in the HIV infected brain [112] up-regulates the expression of CD40 by microglia [113,114]. In AD it has been shown that blood vessels and reactive microglia stain positively for CD40 in *post-mortem *brain tissues. Also in AD brain, aggregates of reactive microglia express CD40 in senile plaques. Up-regulation of CD40 expression by microglia is also seen in a variety of brain lesions without Aβ deposits. They include multiple sclerosis plaques [111] as well as lesions of adrenoleukodystrophy, DRPLA, and ischemic strokes [115]. Aβ was also shown to induce CD40 expression by cultured microglia [116,117] and cultured vascular endothelial cells [116,118][118-120]. HIV-1 induces the latter phenomenon as well [121] It may be the mechanism by which CD40 expression is up-regulated in and around senile plaques in both diseases. However, the results of this study suggest that CD40 expression is induced upon multiple stimuli and that CD40-CD40L interactions are involved rather ubiquitously in activation of microglia and vascular cells.

In regard to HIV-1 neuropathogenesis, a link between CD40 and microglia has been established. Upregulation of CD40 expression has been detected on microglia of HIV-1-infected brain tissues [28]. CD40L was also shown to potentiate the ability of HIV-1 Tat to activate monocytes and microglia leading to the overproduction of inflammatory proteins such as cytokines and chemokines [122].

Furthermore HAART is unable to modulate blood brain barrier (BBB) leakage and inflammation in HAD patients [29,123] in part because it does not reduce the elevated levels of CD40 ligand (CD40L) found in the plasma and CSF of HIV-1-infected patients [122,124]. In further confirmation, other systems [125-127] have shown high levels of sCD40L can modulate CNS inflammation at the level of the BBB.

High levels of soluble CD40L in CSF and plasma of HIV-infected patients with cognitive impairment has been demonstrated as well. Exposure of primary human brain microvascular endothelial cells (BMVECs) to CD40L increased the expression of adhesion molecules intracellular adhesion molecule-1 and vascular cell adhesion molecule-1, which yielded a fourfold increase in monocyte adhesion to BMVECs and stimulated migration across an *in vitro *BBB model [128].

Also central to microglial regulation in HAD, higher levels of sCD40L have been found in the blood and CSF of HIV-infected patients with cognitive impairments compared with HIV-infected subjects without cognitive impairment. Further assays from the same study showed CD40L synergized with HIV-1 Tat to increase TNF-α release from primary human monocytes and microglia, in an NF-κB-dependent manner [122].

Several basic science studies have shown that, during HAD as well as AD, CD40 upregulates the NF-κB pathway, causes hyperactivity in microglia and macrophages, which then produces the release of several neurotoxic compounds such as TNF-α further exacerbating neurodegeneration (for further review see [97,105,106] ). In addition, CD40 activation increases inflammatory responses and decreases the clearance of Aβ. Disrupting CD40 activation by opposing CD40L activity has shown important in improving spatial memory in animal models of AD [117,118,129,130]. Data from our group and others demonstrate the negative regulation of CD40 activation on microglial cells by CD45 [82].

Indeed in contrast to CD40, one cell surface receptor that has been implicated in inhibiting microglial activation is the protein-tyrosine phosphatase (PTP) protein, CD45. It is especially effective at inhibiting microglial activation because its action takes place far upstream from proinflammatory intracellular mediators. We have shown that cross linking CD45 markedly reduces microglial activation resulting from Aβ peptide [131]. Additionally, CD45 inhibits the activation of the p44/42 MAPK pathway; thereby abrogating microglial activation [131]. Mice brains deficient in CD45 have been shown to have increased levels of potentially neurotoxic cytokines such as TNF-α [131]. Taken together, these data seem to suggest that CD45 opposes microglial activation induced by the presence of Aβ peptide.

In addition to its inhibitory effect on Aβ induced microglial activation, CD45 has been shown to inhibit microglial activation induced by several other proinflammatory stimuli [131]. When microglia are incubated with CD45 cross-linking antibodies and LPS, activation was significantly attenuated as evidenced by decreased levels of the neurotoxins TNF-α and NO[131]. This suggests that cross linked CD45 acts to inhibit microglial activation induced by LPS [131]. Other studies also implicate the role of CD45 in negatively regulating cytokine receptor signaling [132,133]. CD45 sufficient macrophages were able to induce greater Aβ clearance, reduced pro-inflammatory (TNF-α) and increased anti-inflammatory (IL-10) cytokines, as well as, potentiate growth factors (TGFβ) in mouse brain. Further, CD45 has also been shown to downregulate NF-kappaB, an important mediator of proinflammatory cytokines and is expressed at a higher rate in HIV infected cells vs. normal cells [134]. Also, matrix metalloproteinase-9 (MMP-9), a protein shown to decrease Aβ plaque formation, was significantly elevated following CD45 administration [135].

In using CD45 to characterize various isoforms of a microglial surface receptor target, our prior studies found that CD45 is able to antagonize CD40L/CD40 mediated-microglia activation [136]. CD45 may perform this function by modulating the production of IL-2, IL-10, and other cytokines and inflammatory factors [97]. Further, co-treatment of microglia with CD40L, in the presence of CD45 activating antibody, results in significant inhibition of microglial TNF-α production through inhibition of p44/42 MAPK activity [82].

In HIV infected patients CD45 expression is decreased. Although this study did not analyze HAD, a lower expression of these proteins on immune cells as well as a higher presence of CD8+ lymphocyte count in HIV+ patients, but not controls, suggests multifactorial immune dysregulation in HIV infected patient; including CD45 dysregulation [137]. Impaired functioning of CD45 is also observed in HIV infected cell cultures. Indeed dysregulated CD45 function likely plays a key role in the inhibition of CD3/CD4 signaling thus contributing to HIV-1 pathogenesis[138]. CD45 antibodies can suppress HIV-infected microglial proliferation, as well as, potently inhibit HIV replication, both *in vitro *and *in vivo*. Microglia that contain CD45 agonist antibody are able to inhibit HIV-1 replication in human cells [139]. Accordingly, HIV infected T-cells display lower levels of CD45 protein; perhaps pointing to a subpopulation susceptible to virus infection or an effect of the virus or viral products on these cells. Indeed, CD45 antibodies have the potential to suppresses neuroinflammation in HAD, AD, and other inflammatory CNS diseases [27,82,131].

In summary, numerous investigations suggest that CD45 plays a key role in regulation of CD40L/CD40-induced microglial activation. This property of microglial CD45 as a regulatory "off switch" for a CD40 promoted, APC-type, M2 type microglia activation phenotype is very likely critical for the prevention and treatment of neurodegeneration found in patients with HAD (Figure 1).

## Abbreviations

Aβ: Amyloid beta/beta amyloid; AD: Alzheimer's disease; APP: Amyloid precursor protein; CNS: Central nervous system; CSF: Cerebrospinal fluid; CD40: Cluster of differentiation 40; CD40L: CD40 ligand; HAD: HIV associated dementia; IFN: Interferon; IL: Interleukin; NSAIDs Non-steriodal anti-inflammatory drugs; sCD40: Soluble CD40; Th: T helper cell; TNF: Tumor necrosis factor; TGF: Transforming Growth Factor; SOCS: Suppressor of cytokine signaling; QUIN: Quinolinic Acid; PAF: Platelet activating factor; Tat: transactivator of transcription

## Competing interests

The authors declare that they have no competing interests.

## Authors' contributions

BG was responsible for the writing of the manuscript, and addressing referee critiques. JS, AC, SR, and JJ were responsible for the initial literature search and first draft one of the review. DO contributed to the generation of Figure 1. JT provided review material for incorporation into the paper regarding the role of CD45 in neurodegeneration in the context of AD. FF provided clinical background regarding HIV and Alzheimer's-type dementias. All authors read and approved the final manuscript.
